# Neuromodulation Through Magnetic Fields Irradiation with AT-04 Improves Hyperalgesia in a Rat Model of Neuropathic Pain via Descending Pain Modulatory Systems and Opioid Analgesia

**DOI:** 10.1007/s10571-023-01430-9

**Published:** 2023-11-07

**Authors:** Tatsuro Kohno, Kaori Takaki, Kaori Kishita, Kazunori Mitsutake, Nozomu Tofuku, Iwao Kishita

**Affiliations:** 1 Anesthesiology and Intensive Care Medicine, International University of Health and Welfare, 852 Hatakeda, Narita City, Chiba 286-0124 Japan; 2 Peace of Mind Co., Ltd, 2-8-6 Tokuo, Kita-Ku, Kumamoto City, Kumamoto 861-5525 Japan

**Keywords:** Neuromodulation, Peripheral nerve injury, Endogenous analgesia, Magnetic fields irradiation, Neuropathic pain

## Abstract

Neuromodulation through magnetic fields irradiation with ait® (AT-04), a device that irradiates a mixed alternating magnetic fields (2 kHz and 83.3 MHz), has been shown to have high efficacy for fibromyalgia and low back pain in our previous clinical trials. The aim of this study was to elucidate the underlying analgesic mechanism of the AT-04 using the partial sciatic nerve ligation (PSL) model as an animal model of neuropathic pain. AT-04 was applied to PSL model rats with hyperalgesia and its pain-improving effect was verified by examining mechanical allodynia using the von Frey method. The results demonstrated a significant improvement in hyperalgesia in PSL model rats. We also examined the involvement of descending pain modulatory systems in the analgesic effects of AT-04 using antagonism by serotonin and noradrenergic receptor antagonists. These antagonists significantly reduced the analgesic effect of AT-04 on pain in PSL model rats by approximately 50%. We also measured the amount of serotonin and noradrenaline in the spinal fluid of PSL model rats using microdialysis during AT-04 treatment. Both monoamines were significantly increased by magnetic fields irradiation with AT-04. Furthermore, we evaluated the involvement of opioid analgesia in the analgesic effects of AT-04 using naloxone, the main antagonist of the opioid receptor, and found that it significantly antagonized the effects by approximately 60%. Therefore, the analgesic effects of AT-04 in PSL model rats involve both the endogenous pain modulation systems, including the descending pain modulatory system and the opioid analgesic system.

**Graphical Abstract:**

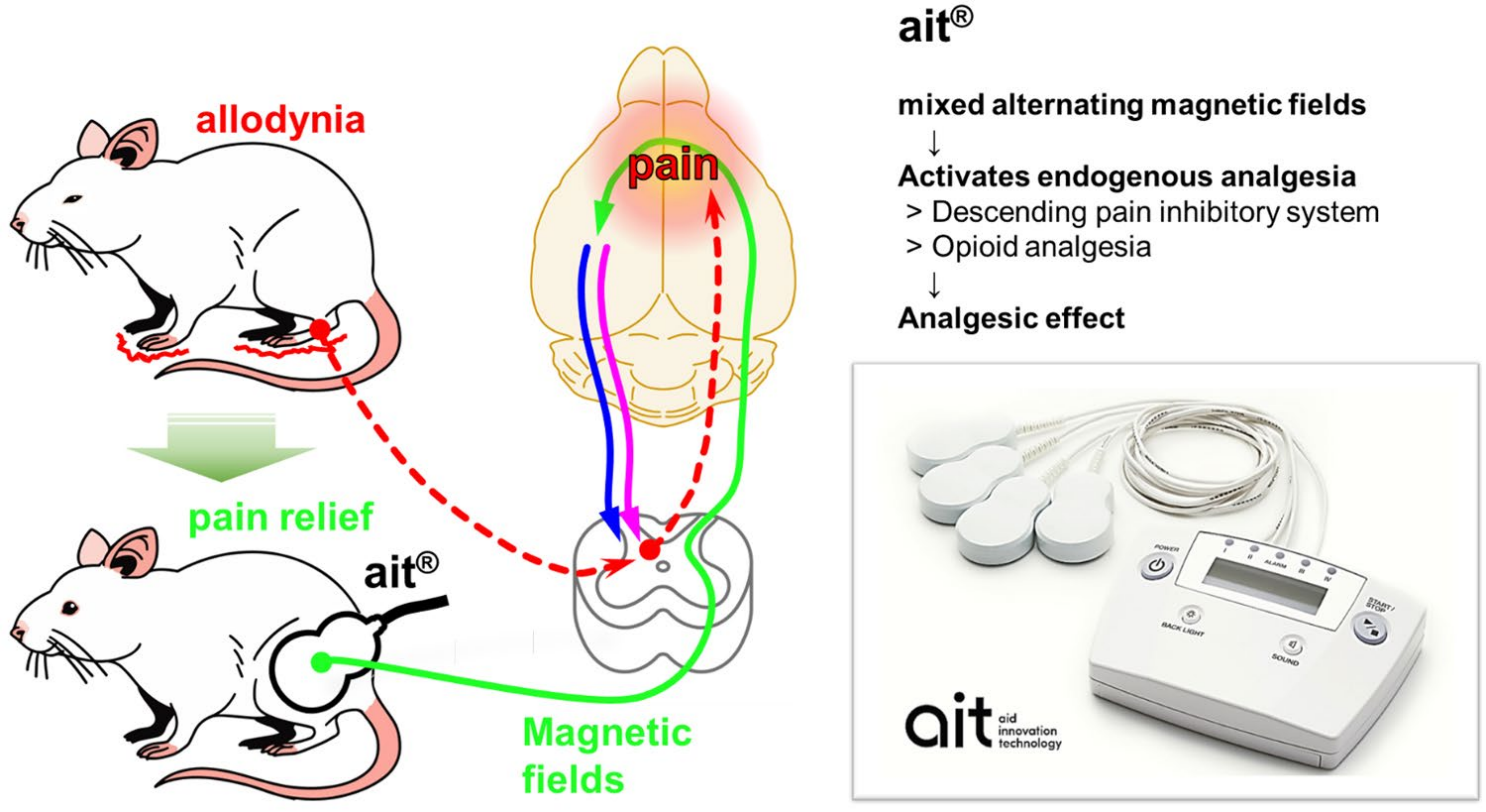

**Supplementary Information:**

The online version contains supplementary material available at 10.1007/s10571-023-01430-9.

## Introduction

Neuropathic pain is defined in international association for the study of pain (IASP) as ‘pain caused by a lesion or disease of the somatosensory nervous system’ (Jensen et al. 2011) and includes all pain resulting from disorders of the somatosensory system of the central and peripheral nervous system. Recently, various drug treatments have been tried in accordance with EBM (evidence-based medicine)-based drug treatment guidelines for neuropathic pain (Attal et al. 2006; Dworkin et al. 2007; Finnerup et al. 2005); however, they may not be sufficiently effective. Moreover, if these drug therapies are inadequate, various other treatments may be tried, including combinations of drugs with different effects, various blocking therapies, and surgical treatment. However, non-pharmacological treatments are not reliably effective, and it is difficult to completely eliminate pain, even with multidisciplinary treatment.

Humans possess an inherent pain suppression system, known as the endogenous analgesic system (Yamamoto and Sakashita 1999). This system includes the descending pain modulation system by serotonergic and noradrenergic neurons, as well as the endogenous opioid analgesia, which controls nociceptive signals from peripheral nerves at the entrance to the central nervous system, specifically the spinal dorsal horn.

However, it has been reported that the endogenous analgesic system is diminished in chronic pain conditions (Basbaum and Fields 1979), such as fibromyalgia (Yarnitsky 2010), and post-herpetic neuralgia (Finnerup et al. 2021), which are classified as neuropathic pain. In these conditions, it is generally believed that neuroplasticity weakens the descending pain modulation system mediated by serotoninergic and noradrenergic neurons, as well as reduces the number and sensitivity of opioid receptors (Kohno et al. 2005), leading to a vulnerability of the endogenous analgesic system. Therefore, the effectiveness of endogenous analgesics for neuropathic pain is considered to be limited.

Despite not being analgesics, antidepressants are effective against neuropathic pain and are used as a first-line treatment for it. This is believed to be because antidepressants inhibit the reuptake of noradrenaline (NA) and serotonin (5-HT) at the synapse, leading to increased NA and 5-HT concentrations in the spinal dorsal horn. This activation of the descending pain inhibiting pathway produces analgesic effects. Additionally, endogenous opioid analgesia is a mechanism in which endogenous opioids, such as β-endorphin and dynorphin, bind to their specific receptors in the central and peripheral nerves and produce morphine-like analgesia (King et al. 2009).

Therefore, patients with refractory chronic pain are typically referred to pain medicine specialists to discuss neuromodulation as a potential treatment option, after initial pain management measures, such as drugs described above, have failed. Neuromodulation refers to the use of medication or technology to modulate pain signaling in the body, with the goal of reducing pain, improving function, and enhancing overall quality of life. Neuromodulation is a therapy that uses implantable or non-implantable devices to reversibly modulate neural activity through electrical and magnetic stimulation, as well as drug administration.

Recent reports have shown that devices stimulating with a combination of alternating magnetic fields and high or low frequencies have shown therapeutic effects on chronic pain in clinical practice (Bagnato et al. 2016; Demirkazik et al. 2019; Aragona et al. 2017). Among these approaches, we directed our attention to the potential that magnetic fields irradiation activates cellular functions, which could result in the regeneration and repair of peripheral sensory nerves (Pleger et al. 2004). Based on this, we aimed to develop a pain treatment device.

In accordance with the gate control theory, two types of nerves mediate stimuli transmission: thin nerve fibers responsible for transmitting pain sensations and thick nerve fibers for transmitting tactile and pressure sensations. In situations involving simultaneous stimuli, the spinal cord—acting as the gateway for sensory transmission to the brain—prioritizes signals from thicker nerves and inhibits signals from thinner nerves, thereby inducing analgesia.

Controlled nerve stimulation relies on weak electrical signals that consequently generate feeble magnetic fields. Typically, the endogenous analgesic mechanism suppresses pain by stimulating nerve fibers involved in pain inhibition through physiological nerve transmission—specifically, physiological magnetic flux density.

Drawing from this understanding, we explored frequencies that could stimulate nerve fibers inhibiting pain with physiological magnetic flux density, leading us to identify a combined alternating magnetic fields of 2 kHz and 83.3 MHz as effective in pain inhibition.

The culmination of our endeavors has led to the development of ait® (AT-04), a portable magnetic fields irradiation device incorporating a combination of mixed alternating magnetic fields—2 kHz (low frequency) and 83.3 MHz (high frequency)—that facilitates the treatment of chronic pain. This device stands as a valuable addition to the realm of neuromodulation therapies.

Our preliminary experiment has been demonstrated that 83.3 MHz showed optimum analgesic effect in a range from 40 to 833 MHz of very high-frequency component (unpublished). It irradiates magnetic fields for 30 min when the probe of AT-04 is applied transcutaneously to the area of peripheral nerve injury. We have previously reported that this magnetic fields irradiation device reduces chronic pain after peripheral nerve injury in rats (Nishi et al. 2004; Shiiba et al. 2012). Furthermore, clinical trials using this magnetic fields irradiation device have shown that it has a high analgesic effect on fibromyalgia patients (using prototype of AT-04, Oka et al. 2020) and on patients with low back pain (using AT-04, in submission). However, the detailed mechanisms underlying the analgesic effect of AT-04 with magnetic fields irradiation are not well understood, as multiple mechanisms are believed to be involved in an integrated manner.

Although many models of neuropathic pain have been reported, PSL model, also called the Seltzer model, was first described by Seltzer et al. in 1990. This model has been prepared by tightly ligating 1/2 to 1/3 of the sciatic nerve in the upper thigh of the rat with 8–0 silk thread (Seltzer et al. 1990). The PSL model shows signs of hyperalgesia, allodynia, and spontaneous pain. This model of neuropathic pain is widely used because it is simple and can be applied to mice. Furthermore, this model is considered more clinically relevant because it exhibits morphine resistance similar to the clinical presentation of neuropathic pain (Han 2003). Thus, the PSL model has been shown to produce chronic neuropathic sensory disorders resembling neuropathic pain in humans.

Therefore, the purpose of this study was to clarify the mechanism of analgesic action of magnetic fields irradiation with AT-04 by focusing on the endogenous pain modulation system, using the PSL model, which has been used in many studies as an animal model of neuropathic pain.

## Methods

### Device

**Fig. 1:**
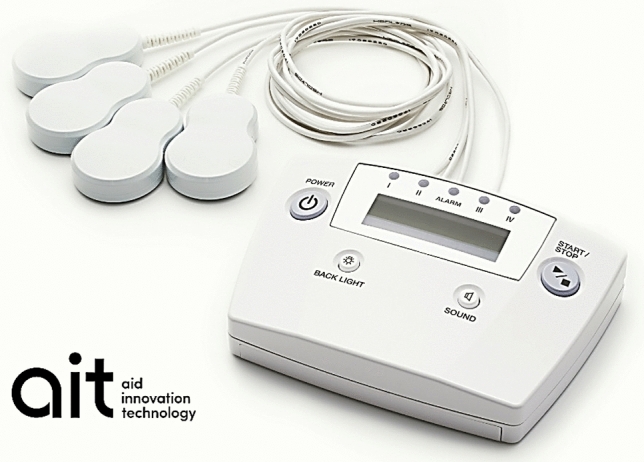
ait® (AT-04), a minimally invasive device developed by Peace of Mind Co., Ltd., that consists of a controller and dual-coil emitter assembly, irradiating a mixed alternating magnetic fields

ait® (AT-04) is a minimally invasive device developed by Peace of Mind Co., Ltd. (Kumamoto, Japan) that consists of a controller and dual-coil emitter assembly (Fig. 1). The dual emitter simultaneously generates alternating magnetic fields at 2 kHz and 83.3 MHz with field strengths of 20–30 µT and 400–700 nT, respectively. A magnetic field has both a magnitude and a direction; an alternating (oscillating) magnetic field exhibits a change in the magnitude and polarity of the field without a change in the direction. The overall energy approximates one-third of terrestrial magnetism. The controller has a timer function designed to discontinue power 30 min after the device is turned on. The sham device has an identical resin case and controller unit but does not generate any alternating magnetic fields.

### Animals

All experiments involving animals were carried out following the ‘Guidelines for the Proper Conduct of Animal Experiments’ established by the Science Council of Japan, at the animal testing facility of Arcrize Japan Co., Ltd. (21,005, 22,006, 22,007, 22,002, Fukuoka, Japan). Male Sprague–Dawley rats weighing 200–230 g were purchased from Nippon Clare Co. (Tokyo, Japan). Rats were kept at room temperature of 23 ± 2 °C under a 12-h light/dark cycle and had free access to food and water. Animal rearing was carried out in 4 animals/cage (W 263 × D 426 × H 202 mm). One animal/cage (W 263 × D 426 × H 202 mm) was also kept during the study.

Pain thresholds were measured prior surgery, and animals that did not respond to the von Frey test (pain threshold; > 60 g) and those that were hyper-responsive (pain threshold; < 6 g) were excluded.

### PSL Model Preparation

The PSL model was established according to previously published methods (Seltzer et al. 1990). Rats were anesthetized with isoflurane by the Univentor 400 anesthesia unit (Univentor, Zejtun, Malta). Intraoperative rat body temperature was measured by a rectal thermometer and maintained at + 37 °C using a BWT-100 Temperature controller (Bio Research Center, Aichi, Japan). The hair of the surgical site in the middle of the rat right thigh was shaved and a 2–3-cm incision was made to expose the sciatic nerve. The sciatic nerve was separated from the surrounding connective tissue and part of the sciatic nerve (approximately 30%) was ligated with 8–0 nylon thread. After sciatic nerve ligation, the incision was sutured. Sham surgery (sham procedure) was performed until the sciatic nerve was exposed and detached from the connective tissue; the sciatic nerve was not ligated and the incision was sutured. After surgery, the rats were individually returned to their cages and left to recover for at least five days until the experiment.

### Grouping

Three days after surgery, pain thresholds were measured using the von Frey test, and the animals with pain thresholds of 5 g or below were selected, and they were assigned to groups to ensure equal distribution of mean and standard error values. However, randomization for random assignment was not conducted in this study.

The sample size for this study was determined through power analysis. A priori power analysis was conducted using an effect size estimate derived from our previous studies, the desired level of statistical power (80%), and the significance level (0.05). The power analysis was performed using G*Power 3.1.9.7 (Heinrich-Heine-Universität Düsseldorf, Germany). However, the results from this preliminary analysis were found to be beyond the physically achievable range. Therefore, in order to establish a realistic sample size, we utilized the maximum feasible upper limit within practical constraints.

### Magnetic Fields Irradiation with AT-04

**Fig. 2:**
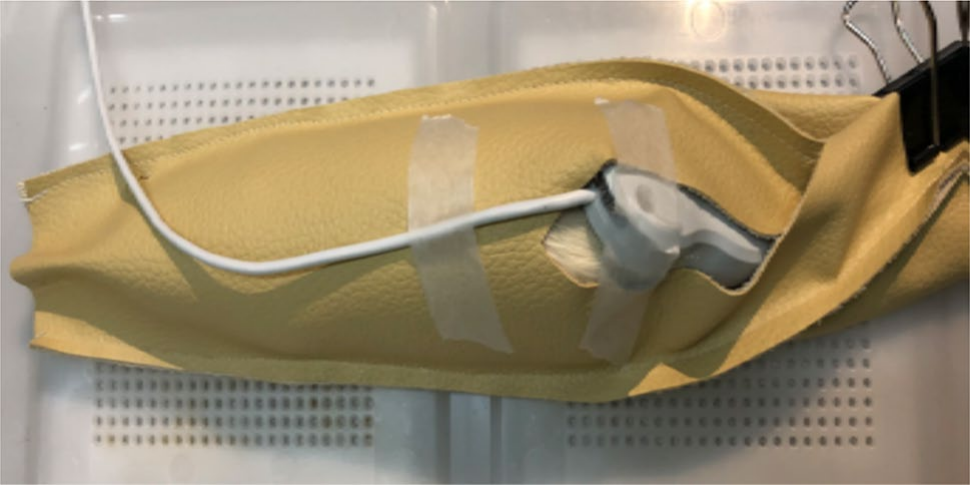
Rat restraint method. The rats were restrained in a restraint bag, and the AT-04 or sham pads were placed near the buttocks and thighs for 30 min of magnetic fields irradiation

After undergoing PSL model surgery, the rats were followed by a recovery period of 5 days. Prior to the first irradiation, their hair was shaved from the irradiated area near the buttocks and thighs. The rats were then placed in a restraint bag (see Fig. 2) and subjected to magnetic fields irradiation twice a day for 30 min per session, with a 6-h interval between the first and second irradiations. This treatment was repeated for several days.

In the evaluation of the analgesic effect of AT-04, AT-04 irradiation was initiated after the postoperative recovery period following surgery, and irradiation with AT-04 for 30 min twice daily was performed for a week (Table 1).

**Table 1:** The protocol for the study

Step/Day		Pre	0	1	2	3	4	5	6	7	8	9	10	11	12	13	14	15
1	PSL surgery		○															
2	Measurement of pain threshold	○				○		○			○				○	○		○
3	1st irradiation with AT-04							○	○	○	○	○	○	○				
4	2nd irradiation with AT-04							○	○	○	○	○	○	○				

The examination was conducted after 5-day postoperative recovery period following PSL surgery and then magnetic fields irradiation with AT-04 for 30 min twice daily for a week was performed. An interval of 6 h was allowed after the first magnetic fields irradiation before conducting the second irradiation. Pain thresholds were evaluated for a total of two weeks, including one week of magnetic fields treatment and one week after treatment

In the evaluation of the antagonistic effects of serotonin and noradrenaline receptor antagonists on the analgesic effect of AT-04, AT-04 irradiation was also initiated after the postoperative recovery period following surgery and irradiation with AT-04 for 30 min twice daily was performed for a week (Table 3).

In the evaluation of the antagonistic effect of the opioid receptor antagonist on the analgesic effect of AT-04, AT-04 irradiation was also initiated after the postoperative recovery period following surgery and irradiation with AT-04 for 30 min twice daily was performed for 3 days (Table 8).

In the microdialysis of serotonin and norepinephrine in spinal cords, AT-04 irradiation was also initiated after the postoperative recovery period following surgery. In the single irradiation group, AT-04 was irradiated once during microdialysis (Table 5). On the other hand, in the repeat irradiation group, AT-04 was also irradiated during microdialysis after 5 days of AT-04 irradiation, following the same protocol as in the single irradiation group (Table 6).

### Drugs Administered

Serotonin receptor antagonist WAY100635 (sc-391296, Santa Cruz Biotechnology, Inc., Dallas, TX, USA), alpha-2 receptor antagonist Yohimbine (Y3125-1G, Sigma-Aldrich Co. LLC, St. Louis, MO, USA), and μ-opioid receptor antagonist Naloxone (144–09411, FUJIFILM Wako Chemicals, Osaka, Japan) were used in this study, with their doses determined based on references (Di Cesare Mannelli et al. 2017; Sakakiyama et al. 2014; Shiiba et al. 2012; Liu et al. 2014). All drugs were dissolved in 0.9% saline and administered intraperitoneally at a dose of 1 mL/kg, 10 min prior to each irradiation with the AT-04 or Sham machine. The drugs or saline were administered twice a day for each irradiation. A control group received the same volume of saline.

### Quantification of Pain Thresholds by Von Frey Test

To quantify pain threshold, we utilized the von Frey test by assessing the escape behavior of the rats in response to stimuli. Prior to the test, the rats were acclimated to the experimental platform with a mesh-like floor surface for approximately 30 min. The von Frey filaments of different diameters (Aesthesio, DanMic Global, LLC, San Jose, CA, USA) were sequentially stimulated to the plantar region of the right hind paw. Each filament was pressed against the paw’s plantar surface for approximately 6 s until it curved. We observed whether escape behavior occurred and recorded the smallest filament stimulus that elicited such a response. This procedure was repeated five times, and the average value was determined as the paw withdrawal threshold (PWT) (Kim et al. 1997; Field et al. 1999; Dowdall et al. 2005).

All behavioral tests were conducted by researchers who maintained the confidentiality of the treatment group. The pain evaluation experiments were carried out at the animal testing facilities of Arcrise-Japan Ltd., in accordance with the Guidelines for the Appropriate Conduct of Animal Experiments set forth by the Science Council of Japan. The pain assessments were performed in a blinded manner, ensuring that the experimenters were unaware of the group assignments of the animals being evaluated.

### Spinal Microdialysis

The microdialysis experiment was performed using anesthetized rats (Nakajima et al. 2012; Ito et al. 2018). Isoflurane anesthesia was performed using the anesthesia unit (Univentor 400, Univentor, Zejtun, Malta). The isoflurane concentration at the time of induction was set to 3–4%, and the isoflurane concentration during the maintenance of anesthesia was set to 1–2%. The body temperature of the rats was measured by a rectal thermometer and maintained at + 37 °C using the temperature controller (BWT-100, Bio Research Center, Aichi, Japan). After shaving the surgical site and exposing the L4 to L5 vertebral arch by cutting, the rats were fixed in a positioning device (SR-7R-HT, Narishige, Tokyo, Japan).

After opening the dura mater with a 30-gauge needle, a microdialysis probe (outer diameter 0.315 mm, membrane length 1 mm, cut-off value 15 kDa, MDP, Arcrize Japan, Japan) was inserted into the right-side root of the rat spinal cord at a 25-degree angle with a micromanipulator (SMM-100, Narishige). The inlet and outlet tubes of the probe were connected to a microinjection pump (55–4143, Harvest Apparatus, Holliston, MA, USA) equipped with a 2.5-mL syringe and an fraction collector (ARJ-MPC11, Arcrize Japan), respectively. The probe was perfused with artificial cerebrospinal fluid (148-mM NaCl, 4-mM KCl, 0.8-mM MgCl2, 1.4-mM CaCl2, 1.2-mM Na2HPO4, 0.3-mM NaH2PO4, pH 7.2) at a flow rate of 0.5 µL/min.

The perfusion fluid was infused 4 h prior to the beginning of irradiation. After 3 h of pre-perfusion, samples were taken every 30 min (15 μL each) using an ARJ-MPC11 fraction collector, starting 1 h before the start of irradiation. The first two samples, including the time 0 sample, were used to determine the baseline levels of 5-HT and NA. The test equipment (G1 and G3 used the Sham machine, G2 and G4 used the AT-04) was then irradiated for 30 min and one sample was taken (15 μL each). Six additional samples were collected every 30 min until 3 h after the end of irradiation (15 μL each). To prevent sample degradation, 0.1-M phosphate buffer (pH 3.0) was added to the recovered samples in a 2:1 sample to acid ratio.

### HPLC Analysis

The concentration of 5-HT and NA was measured using an HPLC-electrochemical detector system (ECD-700, Eicom, Kyoto, Japan) (Ito et al. 2018; Yoshitake et al. 2014). The HPLC system consisted of a pulsed-free low flow rate pump, a degasser, a column oven, and an anodic detector equipped with a graphite electrode subjected to + 0.45 V relative to a silver/silver chloride reference electrode. The samples were injected using an autosampler (AS-4150, Jasco Corp., Tokyo, Japan), with an injection volume of 15 μL. Chromatograms were recorded and processed using data processing software (Clarity, DataApex, Prague, Czech Republic). The separation of 5-HT and NA was performed using a cation-exchange column (CAX, 200 × 2.0 I.D. mm CAX column, Eicom), and the mobile phase consisted of a mixture of 0.1-M phosphate buffer (pH 6.0) and methanol (7:3, v/v) with the addition of 30-mM potassium chloride and 50-mg/L EDTA-2Na. The detection limit (S/N ratio = 3) of 5-HT and NA was 0.5 fmol per 10 μL of sample.

### Statistical Analysis

The raw data for pain threshold was entered into a Microsoft Excel data file. All data were verified prior to analysis, and the mean ± standard deviation (SD) along with the 95% confidence interval (CI) were calculated for each measurement of the pain threshold.

Normality and variance homogeneity was performed ANOVA as well as additional tests, including the Brown–Forsythe test and Bartlett’s test. The comparison of pain thresholds before and after sciatic nerve ligation was performed using *t* tests. The comparison of pain thresholds between different time points and the assessment of group differences for each day were conducted using two-way ANOVA. In the microdialysis test, the comparison of 5-HT and NA at each time within each group was also analyzed using two-way ANOVA. Statistical significance was considered when *P* < 0.05.

The individual data point and analyzed data for each experiment have been documented in each Supplemental Information.

## Results

### Analgesic Effects of Magnetic Fields Irradiation on PSL Model Rat

**Fig. 3:**
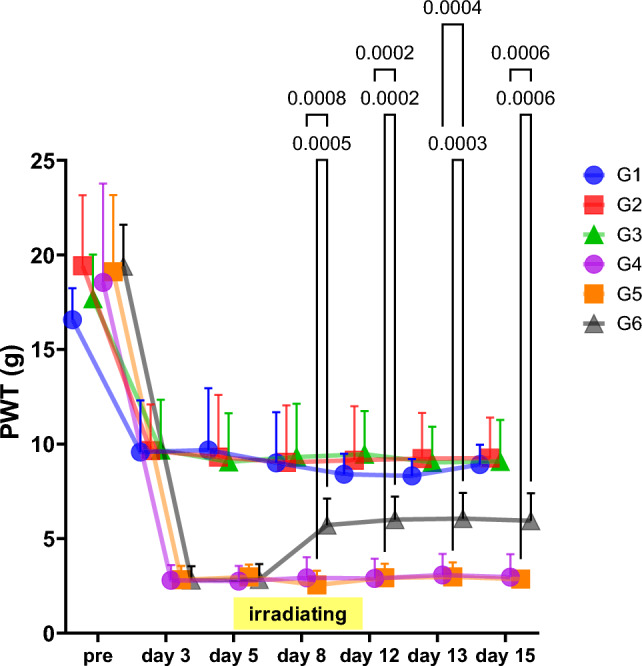
Analgesic Effects of Magnetic fields irradiation on PSL model rat. The figure indicates the paw withdrawal threshold (PWT) on magnetic fields irradiation with AT-04 in PSL surgery rats. The legends indicate the composition of each group as follows: G1: Sham surgery + non-irradiated group, G2: Sham surgery + Sham device-irradiated group, G3: Sham surgery + AT-04-irradiated group, G4: PSL surgery + non-irradiated group, G5: PSL surgery + Sham device irradiation group, and G6: PSL surgery + AT-04 irradiation group. All values were shown as mean ± SD, *n* = 7 rats. Horizontal bar indicates the period of magnetic fields irradiation with sham or AT-04. Statistical significance was analyzed by 2-way ANOVA, p-values (*p* < 0.05 only) were indicated in the graphs

The protocol for the study is presented in Table 1. The analgesic effect of magnetic fields irradiation with AT-04 on allodynia in PSL model rats was evaluated (Table 2, Fig. 3, and SI. 1). The examination was conducted after 5-day postoperative recovery period following PSL surgery and then magnetic fields irradiation with AT-04 for 30 min twice daily for a week was performed. Pain thresholds were evaluated for a total of two weeks, including one week of magnetic fields treatment and one week after treatment. Results were presented as mean ± SD (7 rats per group), along with a 95% confidence interval and the effect size, reported in the Supplemental Information (SI. 1).

**Table 2:** Analgesic effects of magnetic fields irradiation on PSL model rat

			Pre	Day3	Day5	Day8	Day12	Day13	Day15
G1 (Sham surgery + non-irradiated)	Mean		16.57	9.57	9.69	9.00	8.40	8.31	8.91
	SD		1.66	2.74	3.26	2.68	1.08	0.89	1.05
	95% CI	Upper	18.11	12.11	12.70	11.48	9.40	9.14	9.89
		Lower	15.03	7.04	6.67	6.52	7.40	7.49	7.94
G2 (Sham surgery + Sham device irradiated)	Mean		19.43	9.66	9.31	9.03	9.14	9.23	9.26
	SD		3.73	2.44	3.28	3.02	2.86	2.42	2.15
	95% CI	Upper	22.88	11.91	12.35	11.82	11.79	11.46	11.24
		Lower	15.98	7.40	6.28	6.24	6.50	6.99	7.27
G3 (Sham surgery + AT-04 irradiated)	Mean		17.69	9.69	9.06	9.31	9.46	9.03	9.09
	SD		2.34	2.66	2.58	2.82	2.29	1.89	2.19
	95% CI	Upper	19.85	12.15	11.44	11.93	11.57	10.77	11.11
		Lower	15.52	7.22	6.67	6.70	7.34	7.28	7.06
G4 (PSL surgery + non-irradiated)	Mean		18.54	2.79	2.75	2.93	2.89	3.07	2.96
	SD		5.22	0.81	0.80	1.09	1.04	1.11	1.21
	95% CI	Upper	23.37	3.54	3.49	3.93	3.85	4.10	4.08
		Lower	13.71	2.04	2.01	1.92	1.93	2.05	1.84
G5 (PSL surgery + Sham device irradiated)	Mean		19.11	2.82	2.97	2.54	2.92	2.97	2.86
	SD		4.05	0.73	0.65	0.75	0.74	0.76	0.43
	95% CI	Upper	22.86	3.49	3.57	3.24	3.61	3.68	3.25
		Lower	15.37	2.15	2.37	1.85	2.23	2.27	2.46
G6 (PSL surgery + AT-04 irradiated)	Mean		19.40	2.80	2.84	5.71	6.00	6.06	5.94
	SD		2.20	0.73	0.80	1.40	1.22	1.36	1.45
	95% CI	Upper	21.43	3.48	3.58	7.01	7.13	7.31	7.28
		Lower	17.37	2.12	2.10	4.42	4.87	4.80	4.60
2-way ANOVA	G2 vs G3					ns	ns	ns	ns
	G4 vs G6	p-value				0.0008	0.0002	0.0004	0.0006
		Mean Diff				− 2.7890	− 3.1090	− 2.9830	− 2.9830
	G5 vs G6	p-value				0.0005	0.0002	0.0003	0.0006
		Mean Diff				− 3.1710	− 3.0800	− 3.0860	− 3.0860

The table presents the paw withdrawal threshold (PWT) on magnetic fields irradiation with AT-04 in PSL surgery rats. The pain thresholds (g) were indicated as mean ± SD, 95% confidence interval (CI) (7 rats per group). The comparison of pain thresholds between each group at each measurement time point was performed with 2-way ANOVA

Comparison of pain thresholds between groups on Day 3 after surgery showed that the PSL surgery groups (G4, G5, and G6) were significantly lower than in the sham surgery groups (G1, G2, and G3) (*P* < 0.001), confirming the onset of pain in the PSL surgery groups.

When the pain thresholds of the PSL-operated group were determined on Day 8, Day 12, Day 13, and Day 15 when irradiated with the Sham device (G5) and AT-04 (G6), the pain thresholds of G6 on Day 8, Day 12, Day 13, and on Day 15, showing a significant improvement (*P* < 0.001) compared to G5. The results demonstrated the analgesic effect of AT-04 in a sciatic nerve injury model. Furthermore, the analgesic effect persisted after the end of magnetic fields irradiation.

### Noradrenaline and Serotonin Receptor Antagonists Involved in the Analgesic Effects of AT-04 on PSL Model Rat

**Fig. 4:**
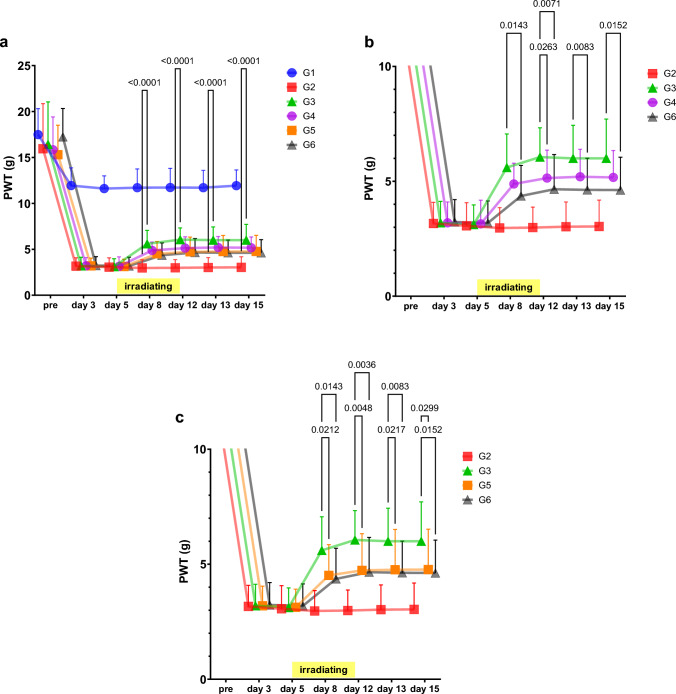
Noradrenaline and serotonin receptor antagonists involved in the analgesic effects of AT-04 on PSL model rat. The figures indicate the paw withdrawal threshold (PWT) in PSL surgery rats on magnetic fields irradiation with AT-04, both with and without antagonists. The legends indicate the composition of each group as follows: G1: sham surgery + solvent + sham device, G2: PSL surgery + solvent + sham device, G3: PSL surgery + solvent + AT-04, G4: PSL surgery + WAY100635 (3 mg/kg) + AT-04, G5: PSL surgery + Yohimbine (3 mg/kg) + AT-04, and G6: PSL surgery + WAY100635 (3 mg/kg) + Yohimbine (3 mg/kg) + AT-04. **a** The evaluation of involvement of 5-HT_1A_ receptor and α_2_-adrenoceptor in the analgesic effect of magnetic fields irradiation with AT-04 yielded significant results (Statistical significance was analyzed by 2-way ANOVA, p-values (*p* < 0.05 only) were indicated in the graphs). **b** The evaluation of the 5-HT_1A_ receptor in the analgesic effect of magnetic fields irradiation with AT-04 showed significant differences compared to G3 (Statistical significance was analyzed by 2-way ANOVA, p-values (*p* < 0.05 only) were indicated in the graphs). **c** The evaluation of α_2_-adrenoceptor in the analgesic effect of magnetic fields irradiation with AT-04 showed significant differences compared to G3 (Statistical significance was analyzed by 2-way ANOVA, *p*-values (*p* < 0.05 only) were indicated in the graphs). Horizontal bar indicates the period of magnetic fields irradiation with sham or AT-04. All values were expressed as mean ± SD with a sample size of *n* = 14 rats

The schedule for the study is presented in Table 3, and alterations of pain thresholds in PSL model rats irradiated with WAY100635 and Yohimbine followed by AT-04 are shown in Fig. 4 and Table 4.

**Table 3:** The protocol for the study

	Step/Day	Pre	0	1	2	3	4	5	6	7	8	9	10	11	12	13	14	15
1	PSL surgery		○															
2	Measurement of pain threshold	○				○		○			○				○	○		○
3	1st drug administration (10 min prior to irradiation with AT-04)							○	○	○	○	○	○	○				
4	1st irradiation with AT-04							○	○	○	○	○	○	○				
5	2nd drug administration (10 min prior to irradiation with AT-04)							○	○	○	○	○	○	○				
6	2nd irradiation with AT-04							○	○	○	○	○	○	○				

The examination was conducted after 5-day postoperative recovery period following PSL surgery and then magnetic fields irradiation with AT-04 for 30 min twice daily for a week was performed. An interval of 6 h was allowed after the first magnetic fields irradiation before conducting the second irradiation. Pain thresholds were evaluated for a total of two weeks, including one week of magnetic fields treatment and one week after treatment. All drugs were dissolved in 0.9% saline and administered intraperitoneally at a dose of 1 mL/kg, 10 min prior to each irradiation with the AT-04 or Sham machine. The drugs or saline were administered twice a day for each irradiation. A control group received the same volume of saline

**Table 4:** Noradrenaline and serotonin receptor antagonists involved in the analgesic effects of AT-04 on PSL model rat

			Pre	Day3	Day5	Day8	Day12	Day13	Day15
G1: sham surgery + solvent + sham device	Mean		17.49	11.94	11.61	11.71	11.73	11.71	11.93
	SD		2.83	1.93	1.38	2.03	2.08	1.88	1.72
	95% CI	Upper	19.12	13.06	12.41	12.89	12.93	12.80	12.92
		Lower	15.85	10.83	10.81	10.54	10.53	10.63	10.94
G2: PSL surgery + solvent + sham device	Mean		15.94	3.17	3.06	2.97	2.99	3.03	3.04
	SD		4.91	0.91	1.01	0.89	0.89	1.08	1.15
	95% CI	Upper	18.78	3.70	3.64	3.48	3.50	3.65	3.70
		Lower	13.11	2.64	2.48	2.46	2.48	2.40	2.38
G3: PSL surgery + solvent + AT-04	Mean		16.43	3.19	3.12	5.60	6.06	6.00	6.00
	SD		4.62	0.93	0.85	1.46	1.27	1.44	1.71
	95% CI	Upper	19.10	3.73	3.61	6.44	6.79	6.83	6.99
		Lower	13.76	2.65	2.62	4.76	5.32	5.17	5.01
G4: PSL surgery + WAY100635 (3 mg/kg) + AT-04	Mean		15.84	3.19	3.15	4.89	5.14	5.20	5.17
	SD		3.57	0.91	1.03	0.90	1.21	1.19	1.17
	95% CI	Upper	17.90	3.72	3.74	5.41	5.84	5.89	5.85
		Lower	13.78	2.67	2.55	4.36	4.44	4.51	4.50
G5: PSL surgery + Yohimbine (3 mg/kg) + AT-04	Mean		15.30	3.19	3.13	4.51	4.73	4.76	4.77
	SD		3.21	0.85	0.79	1.34	1.60	1.75	1.75
	95% CI	Upper	17.15	3.69	3.58	5.29	5.66	5.77	5.78
		Lower	13.45	2.70	2.67	3.74	3.81	3.75	3.76
G6: PSL surgery + WAY100635 (3 mg/kg) + Yohimbine (3 mg/kg) + AT-04	Mean		17.24	3.23	3.19	4.36	4.66	4.63	4.62
	SD		3.08	0.97	0.96	1.33	1.51	1.37	1.43
	95% CI	Upper	19.02	3.79	3.74	5.13	5.53	5.42	5.45
		Lower	15.46	2.67	2.64	3.59	3.79	3.84	3.79
drug antagonism ratio (% of G3)	G4					− 27	− 30	− 27	− 28
	G5					− 41	− 43	− 42	− 42
	G6					− 47	− 46	− 46	− 47
2-way ANOVA in Fig. 4a	G2 vs G3	p-value				< 0.0001	< 0.0001	< 0.0001	< 0.0001
		Mean Diff				− 2.631	− 3.069	− 2.974	− 2.963
2-way ANOVA in Fig. 4b	G3 vs G4	p-value				ns	0.0263	ns	ns
		Mean Diff				0.714	0.914	0.800	0.829
	G3 vs G6	p-value				0.0143	0.0071	0.0083	0.0152
		Mean Diff				1.237	1.400	1.371	1.380
2-way ANOVA in Fig. 4c	G3 vs G5	p-value				0.0212	0.0048	0.0217	0.0299
		Mean Diff				1.086	1.323	1.237	1.231
	G3 vs G6	p-value				0.0143	0.0036	0.0083	0.0152
		Mean Diff				1.237	1.400	1.371	1.380

The table presents the paw withdrawal threshold (PWT) (g) in PSL surgery rats on magnetic fields irradiation with AT-04, both with and without antagonists. The antagonism ratio is expressed as a percentage of G3. The comparison of pain thresholds between each group at each measurement time point was performed using 2-way ANOVA. All values were presented as mean ± SD, 95% confidence interval (CI) (*n* = 14 rats)

After 5-day recovery period from the day of PSL model surgery (Day 0), drug administration and magnetic fields irradiation with AT-04 were performed for 7 days. Pain threshold was determined on 7 times, and on the day of determination of pain threshold and drug administration and magnetic fields irradiation with AT-04, the pain threshold was determined at the beginning, followed by drug administration and magnetic fields irradiation with AT-04. The pain thresholds were indicated as mean ± SD (14 rats per group). Results were presented as mean ± SD (14 rats per group), along with a 95% confidence interval and the effect size, reported in the Supplemental Information (SI. 2).

Comparison of pain thresholds between groups on Day 5 after surgery showed that pain thresholds in the PSL surgery group (G2, G3, G4, G5, and G6) were significantly lower than those in the sham surgery group (G1) (P < 0.001), confirming the development of allodynia in the PSL surgery groups. Furthermore, similarly to the results in Fig. 3, pain thresholds in the PSL surgery rats irradiated with AT-04 (G3, administration of solvent) were significantly increased (*P* < 0.001) compared to the sham device group (G2, administration of solvent), confirming the analgesic effect of AT-04 in PSL rats (Fig. 4a, SI. 2).

Next, to verify the antagonistic effect of WAY100635, a serotonin receptor antagonist, on the analgesic effect of AT-04, we compared the results between different groups. In G4 (administration of WAY100635 only), an antagonistic effect of approximately 30% against the analgesic effect of AT-04 in G3 was observed from Day 8 to Day 15, although it reached statistical significance only on Day 12. On the other hand, G6 (mixed administration of WAY100635 and Yohimbine) exhibited a significant antagonism of approximately 50% (*P* < 0.05) (Fig. 4b, SI. 2).

Moreover, to verify the antagonistic effect of Yohimbine, a noradrenaline receptor antagonist, on the analgesic effect of AT-04, we compared the results between different groups, similarly to Fig. 4b. In G4 (administration of Yohimbine only) showed a significant antagonistic effect of approximately 40% on Day 12 (*P* < 0.05) against the analgesic effect of AT-04 in G3. Furthermore, in G6 (mixed administration of WAY100635 and Yohimbine) exhibited a significant antagonism of approximately 50% (*P* < 0.05) (Fig. 4c, SI. 2).

These results showed that WAY100635 and Yohimbine significantly antagonized against the analgesic effect of AT-04 by approximately 50% and suggested the involvement of noradrenergic and serotonergic receptors in the analgesic effect of AT-04.

### Magnetic Fields Irradiation with AT-04 Increased the Extracellular Levels of 5-HT and NA in the SPINAL Cord of PSL rat

Determination of serotonin and noradrenaline by microdialysis was performed according to the protocol of Tables 5 and 6. Serotonin and noradrenaline in the spinal cord were determined during a single magnetic fields irradiation with AT-04 in G1 (PSL surgery + Sham device irradiated) and G2 (PSL surgery + AT-04 irradiated). Serotonin and noradrenaline in the spinal cord were also determined during single magnetic fields irradiation after repeated magnetic fields irradiation with AT-04 in G3 (PSL surgery + Sham device irradiated) and G4 (PSL surgery + AT-04 irradiated).

**Table 5:** The protocol for the microdialysis experiment of single magnetic fields irradiation

	Step/Day	Pre	0	1	2	3	4	5
1	PSL surgery		〇					
2	Measurement of pain threshold	〇				〇		
3	Microdialysis during irradiation with AT-04 in spinal cord							〇

The examination was conducted after a 5-day postoperative recovery period following PSL surgery. The perfusion fluid was collected during a single magnetic fields irradiation with AT-04 in the spinal cord

**Table 6:** The protocol for the microdialysis experiment of repeat magnetic fields irradiation

	Step/Day	Pre	0	1	2	3	4	5	6	7	8	9	10
1	PSL surgery		〇										
2	Measurement of pain threshold	〇				〇							
3	1st irradiation with AT-04							〇	〇	〇	〇	〇	
4	2nd irradiation with AT-04							〇	〇	〇	〇	〇	
5	Microdialysis during irradiation with AT-04 in spinal cord												〇

The examination was conducted after a 5-day postoperative recovery period following PSL surgery. Magnetic fields irradiation with AT-04 was performed for 30 min twice daily for 5 days. An interval of 6 h was allowed after the first magnetic fields irradiation before conducting the second irradiation. Subsequently, the perfusion fluid was collected during a single magnetic fields irradiation with AT-04 in the spinal cord

The pain thresholds of the rats that underwent PSL surgery used in the experiment showed a significant reduction on day 3 after surgery (*P* < 0.001), confirming the development of allodynia (SI. 3).

**Fig. 5:**
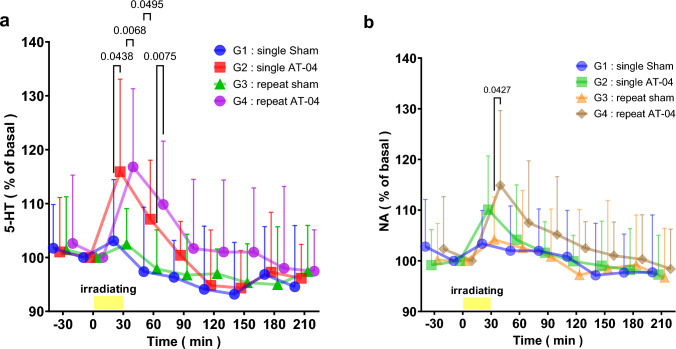
Magnetic fields irradiation with AT-04 increased the extracellular levels of 5-HT and NA in the spinal cord of PSL rat. The figures indicated the changes in extracellular levels of 5-HT and NA in the spinal cord of PSL rats during magnetic fields irradiation with AT-04. **a** The effects of magnetic fields irradiation with AT-04 for 30 min on the extracellular levels of 5-HT, expressed as a percentage of the basal levels at 0 min. The horizontal bar represented the duration of magnetic fields irradiation with sham or AT-04. All values were presented as mean ± SD, *n* = 12 rats. Statistical significance was analyzed by 2-way ANOVA, and *p*-values (*p* < 0.05 only) were indicated in the graphs. **b** The effects of magnetic fields irradiation with AT-04 for 30 min on the extracellular levels of NA, expressed as a percentage of the basal levels at 0 min. The horizontal bar represented the duration of magnetic fields irradiation with sham or AT-04. All values were presented as mean ± SD, *n* = 12 rats. Statistical significance was analyzed by 2-way ANOVA, and *p*-values (*p* < 0.05 only) were indicated in the graphs

The time-dependent manner in extracellular levels of 5-HT and NA in the spinal cord of rats that underwent PSL surgery and were exposed to either Sham or AT-04 devices are shown in Fig. 5 and Table 7. The concentrations of 5-HT and NA (expressed in fmol/10 µL) in each group were calculated as a percentage of the baseline value at 0 min, which was set to 100% and expressed as the mean ± SD (*n* = 12 rats per group), along with a 95% confidence interval and the effect size, reported in the Supplemental Information (SI. 3).

**Table 7:** Magnetic fields irradiation with AT-04 increased the extracellular levels of 5-HT and NA in the spinal cord of PSL rat

				Time (min)								
					basal	irradiating						
				− 30	0	30	60	90	120	150	180	210
5-HT ratio (%)	G1 (Single Sham)	Mean		101.72	100.00	103.08	97.38	96.33	94.11	93.19	96.85	94.57
		SD		8.10	0.00	11.37	11.95	6.84	11.71	9.62	8.89	11.35
		95% CI	Upper	106.86	100.00	110.30	104.98	100.68	101.55	99.30	102.50	101.78
			Lower	96.57	100.00	95.85	89.79	91.99	86.67	87.08	91.20	87.35
	G2 (Single AT-04)	Mean		101.04	100.00	115.91	107.11	100.42	94.80	94.33	97.27	96.14
		SD		10.09	0.00	17.17	10.93	6.27	10.31	6.95	11.11	6.28
		95% CI	Upper	107.45	100.00	126.82	114.05	104.40	101.35	98.75	104.32	100.13
			Lower	94.63	100.00	105.00	100.16	96.43	88.25	89.92	90.21	92.15
	G3 (Repeat Sham)	Mean		101.43	100.00	102.46	97.88	96.75	97.00	95.33	94.96	97.41
		SD		9.83	0.00	6.63	7.25	7.57	4.58	7.21	10.75	8.54
		95% CI	Upper	107.68	100.00	106.67	102.48	101.56	99.91	99.91	101.79	102.84
			Lower	95.19	100.00	98.25	93.27	91.94	94.09	90.74	88.13	91.98
	G4 (Repeat AT-04)	Mean		102.60	100.00	116.82	109.84	101.68	101.03	100.98	98.01	97.46
		SD		12.70	0.00	14.50	11.74	12.83	13.35	11.93	15.19	7.66
		95% CI	Upper	110.67	100.00	126.03	117.30	109.82	109.51	108.56	107.66	102.33
			Lower	94.53	100.00	107.60	102.38	93.53	92.54	93.39	88.36	92.59
	2-way ANOVA	Single G1 vs G2	*p*-value			0.0438	0.0495	ns	ns	ns	ns	ns
			Mean Diff			− 12.83	− 9.73	− 4.08	− 0.69	− 1.14	− 0.42	− 1.58
		Repeat G3 vs G4	*p*-value			0.0068	0.0075	ns	ns	ns	ns	ns
			Mean Diff			− 14.36	− 11.97	− 4.93	− 4.03	− 5.65	− 3.05	− 0.05
NA ratio (%)	G1 (Single Sham)	Mean		102.81	100.00	103.38	101.98	101.97	100.81	97.13	97.68	97.74
		SD		9.28	0.00	6.58	8.84	8.26	9.14	10.28	7.59	11.30
		95% CI	Upper	108.71	100.00	107.55	107.60	107.22	106.62	103.66	102.51	104.92
			Lower	96.91	100.00	99.20	96.37	96.72	95.00	90.60	92.86	90.56
	G2 (Single AT-04)	Mean		99.14	100.00	110.08	104.16	101.61	99.90	99.03	98.18	97.30
		SD		7.02	0.00	10.60	10.81	6.10	5.12	8.78	8.18	7.75
		95% CI	Upper	103.60	100.00	116.82	111.03	105.49	103.15	104.61	103.37	102.22
			Lower	94.68	100.00	103.35	97.29	97.73	96.65	93.46	92.98	92.38
	G3 (Repeat Sham)	Mean		100.13	100.00	104.18	102.53	100.83	97.19	98.48	99.18	96.68
		SD		7.22	0.00	8.43	11.35	9.38	12.93	13.43	9.84	9.72
		95% CI	Upper	104.72	100.00	109.54	109.74	106.78	105.41	107.01	105.44	102.86
			Lower	95.55	100.00	98.83	95.31	94.87	88.98	89.94	92.93	90.51
	G4 (Repeat AT-04)	Mean		102.59	100.00	114.88	107.70	105.37	102.69	101.23	100.51	98.58
		SD		10.61	0.00	14.75	12.01	11.25	5.03	6.46	8.65	7.61
		95% CI	Upper	108.90	100.00	124.25	115.26	112.44	105.81	105.24	105.88	103.39
			Lower	95.80	100.00	105.53	99.72	97.88	99.15	96.81	94.72	93.39
	2-way ANOVA	Single G1 vs G2	*p*-value			ns	ns	ns	ns	ns	ns	ns
			Mean Diff			− 6.71	− 2.18	0.36	0.91	− 1.90	− 0.49	0.44
		Repeat G3 vs G4	*p*-value			0.0427	ns	ns	ns	ns	ns	ns
			Mean Diff			− 10.71	− 4.97	− 4.33	− 5.29	− 2.55	− 1.12	− 1.71

The table presented the changes in extracellular levels of 5-HT and NA in the spinal cord of PSL rats during magnetic fields irradiation with AT-04. The levels were expressed as a percentage of the basal levels and were analyzed by 2-way ANOVA to compare the AT-04 group with the sham group. All values were presented as mean ± SD, 95% confidence interval (CI) *n* = 12 rats

The basal values of extracellular 5-HT and NA in the spinal cord of rats used in the experiments at 0 min were 2.26 ± 0.13 fmol/10 µL and 1.79 ± 0.09 fmol/10 µL in G1 (Sham device single irradiated), 2.05 ± 0.14 fmol/10 µL and 1.64 ± 0.08 fmol/10 µL in G2 (AT-04 device single irradiated), 2.47 ± 0.14 fmol/10 µL and 1.80 ± 0.10 fmol/10 µL in G3 (Sham device repeat irradiated), and 2.20 ± 0.13 fmol/10 µL and 1.73 ± 0.05 fmol/10 µL in G4 (AT-04 repeat irradiated), respectively.

The change ratio of extracellular 5-HT and NA in the spinal cord collected during a single irradiation with AT-04 in time point at 30 min were 115.9 ± 4.96% and 110.1 ± 3.06%, respectively (Fig. 5a, b). The concentration of 5-HT was significantly increased (*P* < 0.05) in the AT-04 irradiation group (Fig. 5a, SI. 3). However, NA concentration tended to increase compared to the Sham device irradiation group (Fig. 5b, SI. 3), although this difference was not statistically significant. Furthermore, the 5-HT concentration after a single irradiation of AT-04 showed a significant increase in time point at 60 min (*P* < 0.05) up to 30 min after the end of irradiation compared to the Sham device irradiation group.

The change ratio of extracellular levels of 5-HT and NA in the spinal cord collected during irradiation (30 min AT-04 irradiation) after repeated AT-04 irradiation were 116.8 ± 4.18% and 114.9 ± 4.26%, respectively (Fig. 5a, b, SI. 3). The 5-HT and NA concentrations were significantly increased (5-HT; *P* < 0.01, NA; *P* < 0.05) compared to the Sham irradiation group. Moreover, 5-HT and NA concentrations after repeated irradiation of AT-04 were 109.9 ± 3.38% and 107.7 ± 3.47% in time point at 60 min (30 min after the end of irradiation), respectively. The 5-HT concentration increased significantly (*P* < 0.05) up to 30 min after irradiation compared to the Sham irradiation group. On the other hand, NA concentrations tended to increase, although the difference was not statistically significant compared to the Sham irradiation group.

### µ-Opioid Receptor Antagonists Involved in the Analgesic Effects of AT-04 on PSL Model Rat

Next, the involvement of endogenous opioids, which were one of the endogenous pain suppression systems, in the analgesic effect of AT-04 was examined in a drug antagonism test using naloxone, an opioid receptor antagonist. The experiment was conducted following the procedure outlined in Table 8.

**Table 8:** The protocol for the study

	Step/Day	Pre	0	1	2	3	4	5	6	7	8
1	PSL surgery		〇								
2	Measurement of pain threshold	〇				〇		〇			〇
3	1st drug administration (10 min prior to irradiation with AT-04)							〇	〇	〇	
4	1st irradiation with AT-04							〇	〇	〇	
5	2nd drug administration (10 min prior to irradiation with AT-04)							〇	〇	〇	
6	2nd irradiation with AT-04							〇	〇	〇	

The examination was conducted after a 5-day postoperative recovery period following PSL surgery. Subsequently, magnetic fields irradiation with AT-04 was performed for 30 min twice daily for 3 days. An interval of 6 h was allowed after the first magnetic fields irradiation before conducting the second irradiation. Pain thresholds were evaluated before irradiation with AT-04. The μ-opioid receptor antagonist Naloxone was dissolved in 0.9% saline and administered intraperitoneally at a dose of 4 mL/kg, 10 min prior to each irradiation with the AT-04 or Sham machine. The drugs or saline were administered twice a day for each irradiation. The control group received the same volume of saline

**Fig. 6:**
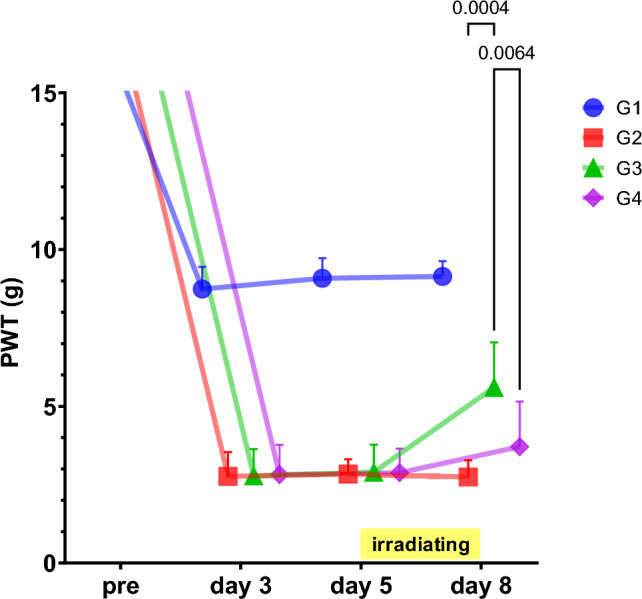
*µ*-opioid receptor antagonists involved in the analgesic effects of AT-04 on PSL model rat. The figure indicates the paw withdrawal threshold (PWT) in PSL surgery rats on magnetic fields irradiation with AT-04, both with and without antagonists. The legends indicate the composition of each group as follows: G1: sham surgery + solvent + sham device, G2: PSL surgery + solvent + sham device, G3: PSL surgery + solvent + AT-04, and G4: PSL surgery + naloxone (4 mg/kg) + AT-04. Horizontal bar indicates the period of magnetic fields irradiation with sham or AT-04. All values were shown as mean ± SD, *n* = 7 rats. Statistical significance was analyzed by 2-way ANOVA, and *p*-values (*p* < 0.05 only) were indicated in the graphs

The alteration of pain thresholds in PSL rats exposed to magnetic fields irradiation with AT-04 after naloxone administration are shown in Fig. 6 and Table 9. Pain thresholds were shown as mean ± SD (7 rats per group), along with a 95% confidence interval and the effect size, reported in the Supplemental Information (SI. 4).

**Table 9:** *µ*-opioid receptor antagonists involved in the analgesic effects of AT-04 on PSL model rat

			Pre	Day3	Day5	Day8
G1: sham surgery + solvent + sham device	Mean		19.00	8.74	9.09	9.14
	SD		4.63	0.71	0.64	0.49
	95% CI	Upper	23.28	9.40	9.68	9.59
		Lower	14.72	8.09	8.49	8.69
G2: PSL surgery + solvent + sham device	Mean		18.63	2.77	2.84	2.74
	SD		3.26	0.78	0.47	0.54
	95% CI	Upper	21.64	3.48	3.27	3.24
		Lower	15.61	2.05	2.41	2.25
G3: PSL surgery + solvent + AT-04	Mean		17.54	2.78	2.90	5.60
	SD		3.86	0.85	0.88	1.44
	95% CI	Upper	21.11	3.57	3.71	6.93
		Lower	13.97	1.99	2.09	4.27
G4: PSL surgery + naloxone (4 mg/kg) + AT-04	Mean		20.06	2.82	2.88	3.71
	SD		4.99	0.95	0.77	1.44
	95% CI	Upper	24.67	3.70	3.59	5.04
		Lower	15.44	1.95	2.17	2.39
drug antagonism ratio (% of G3)	G4					− 66
2-way ANOVA	G2 vs G3	p-value				0.0004
		Mean Diff				− 2.857
	G3 vs G4	p-value				0.0064
		Mean Diff				1.886

The table presents the paw withdrawal threshold (PWT) in PSL surgery rats on magnetic fields irradiation with AT-04, both with and without antagonists. The antagonism ratio was expressed as a percentage of G3. The comparison of pain thresholds between each group at each measurement time point was performed using 2-way ANOVA. All values were presented as mean ± SD, 95% confidence interval (CI) (*n* = 7 rats)

The pain thresholds of the rats that underwent PSL surgery used in the experiment showed a significant reduction on day 3 after surgery (*P* < 0.001), confirming the development of allodynia. On the other hands, the pain thresholds on day 8 after magnetic fields irradiation with AT-04 for 3 days from Day 5 after surgery were compared between G2 (PSL surgery + solvent + Sham device irradiation) and G3 (PSL surgery + solvent + AT-04 irradiation) and a significant increase in G3 (*P* < 0.001) confirmed the analgesic effect of AT-04.

Moreover, to verify the antagonistic effect of naloxone, a µ-opioid receptor antagonist, on the analgesic effect of AT-04 on Day 8, we compared the results between G3 (PSL surgery + solvent + AT-04 irradiation) and G4 (PSL surgery + naloxone (4 mg/kg) + AT-04 irradiation). The results showed a significant antagonistic effect of naloxone of approximately 60% in G4, confirming the involvement of µ-opioid receptors in the analgesic effect of AT-04 (*P* < 0.01) (Fig. 6 and Table 9, and SI. 4).

## Discussion

This study aimed to elucidate the analgesic mechanism of our developed low-powered portable alternating magnetic fields device, AT-04, which features two different frequency modes: 2 kHz (low frequency) and 83.3 MHz (high frequency). The results demonstrated that AT-04 significantly improved allodynia in PSL rats. Furthermore, the analgesic effect of AT-04 was antagonized by specific antagonists targeting 5-HT, NA, and μ-opioid receptors. Additionally, the levels of 5-HT and NA in the spinal cord of PSL rats were found to increase with the irradiation of AT-04. Collectively, these findings provide evidence that the analgesic effect of AT-04 is attributed to the activation of endogenous analgesia.

In our preliminary study, we evaluated the analgesic effects of AT-04 by applying magnetic fields irradiation at three different sites (near the spinal cord, near the buttocks and thigh, and near the abdomen) in PSL model rats and assessed the pain suppression effects. Although detailed data were not shown, we observed analgesic effects of AT-04 not only in the vicinity of the affected area (ligation site) near the buttocks and thigh but also in the vicinity of the spinal cord and abdomen. Furthermore, in non-clinical trials on animals modeling chronic pain as a fibromyalgia model induced by repeated cold stress, which is known to decrease the endogenous pain control system (Itomi et al. 2016), AT-04 has demonstrated a significant analgesic effect not only in the nerve ligation model but also in the fibromyalgia model (data not shown).

Based on these results, including the preliminary study, we speculate that the analgesic effect of AT-04 with magnetic fields irradiation is achieved by activating the endogenous pain control system in neuropathic pain.

Furthermore, the analgesic effect of AT-04 is suggested to be attributed to the activation of endogenous analgesia by neuromodulation-induced neural plasticity, as evidenced by the sustained effect of magnetic fields irradiation on mechanical allodynia after treatment, as shown in Fig. 3 and Fig. 4. On the other hand, a pharmacological antagonism test using naloxone, a major opioid receptor antagonist, revealed that naloxone partially blocked the analgesic effect of AT-04 by approximately 60%. However, beta-endorphin, one of the endogenous opioids, did not show an increase in the gray matter of the brain (data not shown). Nevertheless, our preliminary data confirmed the upregulation of opioid receptors in the brain of a repeated cold stress mouse model of fibromyalgia, as well as in microglial cell lines (data not shown).

Overall, these findings, including the results of the preliminary study, provide support for the discussion of the current findings.

Previous clinical trials using this device have shown a high analgesic effect on patients with fibromyalgia (using the AT-02 prototype of AT-04, Oka et al. 2020) and low back pain (using AT-04, in submission).

In neuropathic pain, the neural circuits that inhibit pain are impaired by damage or inflammation and the plasticity of neural circuits that regulate pain is abnormal, resulting in a decrease in the analgesic effects of descending pain inhibitory systems and endogenous analgesics (Costigan et al. 2009). Exercise therapy (Stagg et al. 2011), pharmacotherapy (Dworkin et al. 2007), and electrotherapy (DeSantana et al. 2009) are among the methods used to activate endogenous analgesia in neuropathic pain. Moderate exercise is known to promote the activation of the endogenous opioid system and alleviate neuropathic pain, but exercise programs need to be tailored to the individual patient’s abilities and symptoms.

On the other hand, pharmacotherapy with antidepressants, anticonvulsants, and other drugs is also used to relieve neuropathic pain, but the potential for ineffective treatment and significant side effects should be taken into consideration. Furthermore, neuromodulation of electrical therapies such as transcutaneous electrical nerve stimulation (TENS) and spinal cord stimulation (SCS) is believed to alleviate neuropathic pain by activating low-threshold afferent fibers (Sdrulla et al. 2015), although their mechanisms of action on other nerve fibers are not yet fully understood. Furthermore, the mechanical stimulation of peripheral nerves has demonstrated significant biological changes in neuropathic pain/inflammation biomarkers and nerve regeneration processes in both preclinical and clinical models (Carta G et al., 2022; Ellis R., 2022; Zhu GC et al., 2022).

Magnetic fields administration by AT-04 could be used alone or in combination with these therapies, including other non-pharmacological interventions providing physical stimuli that are available to treat neuropathic pain. This approach may potentially result in synergistic effects for alleviating neuropathic pain. The underlying mechanisms of neuropathic pain are diverse, making it essential to establish effective and individualized treatment strategies.

Although alternating magnetic fields therapy has been reported as effective in improving pain for some time, the specific type of pain for which alternating magnetic fields therapy is effective may vary depending on the type, cause, and degree of pain, making it important to select an appropriate treatment method. Furthermore, recent reports have shown that devices stimulating with a combination of alternating magnetic fields and low frequencies or high frequencies have shown therapeutic effects on chronic pain in clinical practice (Bagnato et al. 2016; Demirkazik et al. 2019; Aragona et al. 2017).

In 1965, Melzack and Wall proposed the Gate Control Theory (Melzack and Wall 1965), which explains the mechanism of pain sensation transmission to the brain. According to this theory, the perception of pain is modulated by a “gate” located in the spinal cord that regulates the number of signals passing through it. Pain signals conveyed by C fibers and Aδ fibers pass through the gate, resulting in pain perception. In contrast, when pain-suppressing Aβ fibers are stimulated simultaneously, the gate is closed, leading to pain relief. This gate control mechanism is one of the endogenous pain control systems that physiologically alleviate pain.

The large diameter, myelinated (Aβ) primary afferents have a low mechanical threshold and transmit non-painful, tactile, and proprioceptive information to the spinal cord. Spinal cord stimulation (SCS) is believed to exert its analgesic effects by stimulating Aβ fibers through the stimulation of the dorsal columns of the spinal cord, resulting in retrograde inhibition of synaptic transmission at the dorsal horn level (Linderoth and Foreman 1999, 2017). It is hypothesized that SCS promotes the release of GABA and acetylcholine (Schechtmann et al. 2008), leading to the suppression of neuronal excitability in the projecting neurons and providing pain relief. Additionally, activation of descending pain modulatory systems, such as the serotonin and noradrenaline systems, has also been reported as a mechanism of analgesic action (Song et al. 2011).

This study demonstrated that the analgesic effect of AT-04 is attributed to the activation of endogenous analgesia. Aβ fibers are primarily activated by mild physical stimuli, such as touch, vibration, and pressure, which occur in peripheral tissues, such as the skin and muscles. Therefore, it is possible that the magnetic fields irradiation of Aβ fibers in the periphery by AT-04 may activate endogenous analgesia through neural plasticity. Although this study does not directly prove the involvement of Aβ fibers, we plan to investigate this further.

Finally, patients with chronic pain that is unresponsive to treatment are expected to face difficulties in visiting hospitals. This device, anticipated to become more widely used, allows patients to receive treatment at home, thereby minimizing the burden on the patient. Additionally, it is non-invasive, ensuring a comfortable and convenient experience for the users.

### Supplementary Information

Below is the link to the electronic supplementary material.

Supplementary file1 (XLSX 76 kb)

Supplementary file2 (XLSX 110 kb)

Supplementary file3 (XLSX 140 kb)

Supplementary file4 (XLSX 39 kb)

## Data Availability

Enquiries about data availability should be directed to the authors.
